# Anticancer Properties of Distinct Antimalarial Drug Classes

**DOI:** 10.1371/journal.pone.0082962

**Published:** 2013-12-31

**Authors:** Rob Hooft van Huijsduijnen, R. Kiplin Guy, Kelly Chibale, Richard K. Haynes, Ingmar Peitz, Gerhard Kelter, Margaret A. Phillips, Jonathan L. Vennerstrom, Yongyuth Yuthavong, Timothy N. C. Wells

**Affiliations:** 1 Medicines for Malaria Venture (MMV), Geneva, Switzerland; 2 St. Jude Children's Research Hospital, Memphis, Tennessee, United States of America; 3 Department of Chemistry and Institute of Infectious Disease and Molecular Medicine, University of Cape Town, Rondebosch, South Africa; 4 Centre of Excellence for Pharmaceutical Sciences, North-West University, Potchefstroom, South Africa; 5 Oncotest GmbH, Freiburg, Germany; 6 Department of Pharmacology, University of Texas Southwestern Medical Center, Dallas, Texas, United States of America; 7 Department of Pharmaceutical Sciences, Nebraska Medical Center, Omaha, Nebraska, United States of America; 8 BIOTEC, National Science and Technology Development Agency, Thailand Science Park, Pathumthani, Thailand; Royal Tropical Institute, The Netherlands

## Abstract

We have tested five distinct classes of established and experimental antimalarial drugs for their anticancer potential, using a panel of 91 human cancer lines. Three classes of drugs: artemisinins, synthetic peroxides and DHFR (dihydrofolate reductase) inhibitors effected potent inhibition of proliferation with IC_50_s in the nM- low µM range, whereas a DHODH (dihydroorotate dehydrogenase) and a putative kinase inhibitor displayed no activity. Furthermore, significant synergies were identified with erlotinib, imatinib, cisplatin, dasatinib and vincristine. Cluster analysis of the antimalarials based on their differential inhibition of the various cancer lines clearly segregated the synthetic peroxides OZ277 and OZ439 from the artemisinin cluster that included artesunate, dihydroartemisinin and artemisone, and from the DHFR inhibitors pyrimethamine and P218 (a parasite DHFR inhibitor), emphasizing their shared mode of action. In order to further understand the basis of the selectivity of these compounds against different cancers, microarray-based gene expression data for 85 of the used cell lines were generated. For each compound, distinct sets of genes were identified whose expression significantly correlated with compound sensitivity. Several of the antimalarials tested in this study have well-established and excellent safety profiles with a plasma exposure, when conservatively used in malaria, that is well above the IC_50_s that we identified in this study. Given their unique mode of action and potential for unique synergies with established anticancer drugs, our results provide a strong basis to further explore the potential application of these compounds in cancer in pre-clinical or and clinical settings.

## Introduction

Over the past two decades, numerous studies have identified antitumor activities of malaria drugs. Nearly all these studies focused on artemisinin derivatives, which are based on natural sesquiterpene lactones with a 1,2,4-trioxane ring system. Originally isolated from *Artemisia* plants, this scaffold currently represents a cornerstone of the fight against malaria [1]. Artemisinin itself and its derivatives artesunate (ART), arteether, artemether and dihydroartemisinin (DHA) are, variously formulated, used in malaria. In addition, intense activity is aimed at exploring additional, synthetic peroxides. A considerable motivation for the interest in artemisinins in additional indications is their excellent, well-established safety profile.

The vast majority of studies with artemisinins for use in cancer (188 to date) involve *in vitro* and *in vivo* experiments aimed at establishing the drug's mode of action and potential for synergy with established cancer drugs ([2]–[12]; see also the recent review [13]). By contrast, only a few clinical studies -mostly anecdotal findings from single cases, and one formal trial- have been performed [[14]–[20]; see [21] for a recent review of clinical uses], reporting modest improvement in patients with advanced non-small lung cancer. In light of the vast preclinical literature on anticancer properties of artemisinins and their excellent, well-established safety profile it is surprising that there are not more reports, or more widespread off-label use of artemisinins for cancer. As was pointed out recently [21] one issue with artemisinins is their short half-life in patients and variability in drug exposure between patients (eg [22], [23]) and over time [24], [25]. These problems are no major obstacle for eliminating *Plasmodium* parasites in malaria patients over a three-day cure, but may prevent efficient inhibition of metastasis-associated angiogenesis, if that were the principal mode of action for artemisinins' use in cancer. The major issue had been the lack of registration for any indication in the US and the general lack of clinical grade material (produced under GMP conditions). There has been a recent step forward here with the WHO prequalification of artesunate for injection produced in China by Guilin in 2010, confirming that production is at internationally recognized GMP standards, and this product is available in Europe under special conditions. Sigma-Tau, as part of their collaboration with the US Army, has obtained an orphan drug designation for artesunate use against malaria from the United States FDA, and with an FDA filing planned for the first quarter in 2014 (Pietro Grossi, *pers. comm*.). This should pave the way to the registration of an artesunate for injection in the USA early in 2014.

In spite of extensive efforts and significant progress, the mode of action of artemisinins in eliminating intra-erythrocytic *Plasmodium* parasites, and their activity in cancer is still incompletely understood. For use in malaria, structure-activity relationships among analogs implicate the 1,2,4-trioxane peroxide pharmacophore as critical for artemisinins' function [26]. One potential mechanism comes from the demonstration that interaction with free Fe^2+^ or heme [27], [28] _ENREF_36 triggers a chemical cascade that generates multiple, toxic reactive oxygen species (ROS; reviewed in [29]). Alternatively, or in parallel, artemisinins bind to heme and interfere with *Plasmodium*'s critical detoxification of heme whereby toxic hematin is polymerized into an insoluble crystalline form of β-hematin called hemozoin, also known as malaria pigment. Three anticancer mechanisms have been identified for artemisinins: A few reports suggest that artemisinins may steer the immune system from a Th2 to a Th1-dominated anticancer response with suppression of the T_reg_ population [8], [30]. Second, many studies find that artemisinins can interfere with angiogenesis [31]–[37] by repression of VEGF and Ang-1 secretion and interfering with chorioallantoic membrane neovascularization. However, the vast majority of publications demonstrate that artemisinins specifically induce apoptosis in a variety of tumor cell lines (see [13] for a review). Molecular analysis indicates that apoptosis is induced through the intrinsic pathway (eg [12], [38], [39]), involving Bcl-2, Bcl-X_L_, Bak/Bax, mitochondrial release of cytochrome c, loss of mitochondrial membrane potential and activation of caspases 9 and −3 (but not caspase 8, which mediates the extrinsic/TNFR-related pathway). Again, activation with Fe^2+^ appears to play a major role: preloading cancer cells with iron or iron-saturated holo-transferrin significantly potentiates the induction of apoptosis (eg [40]–[44]). The enhanced metabolic need for iron in cancer cells is well-established, and the up-regulated transferrin receptor is being investigated as a means to specifically target cancer cells [45], [46]. Artemisinins appear to be directly activated by heme [47], as illustrated by the finding that compounds that bind mitochondrial heme also show good cytotoxicity [48]. The critical role of mitochondrial function in mediating the cytotoxicity of artemisinin was elegantly demonstrated in a yeast system [49]. Importantly, this mode of action is distinct from existing anticancer drugs. Consequently, artemisinins have a great potential to synergize with established treatments both *in vitro* and *in vivo* (eg [40], [50]–[52]), even more importantly, multi-drug resistant cancers retain sensitivity towards artemisinins [53]–[56].

In order to explore the potential use of antimalarials in cancer it is important to further link their mode of action with the critical signaling pathways that drive cancer cell proliferation. This enables us to move beyond a simple and antiquated categorization of cell lines predominantly by the organ from which they originated. Linked to this, it is important to explore what the synergies with current anti-cancer drugs will be. The primary focus of this study was to test the semisynthetic artemisinins artesunate and its active metabolite dihydroartemisinin, the semisynthetic N-thiomorphilino derivative, artemisone (BAY 44-9585 [57]) and two synthetic peroxides, OZ277 (also called arterolane) and OZ439. As a positive control, we also tested two anti-parasite dihydrofolate reductase inhibitors, pyrimethamine (recently shown to induce apoptosis in melanoma cells [58]) and P-218 [59], which were anticipated to show some activity against a human cancer cell panel. In addition we tested the highly selective parasite dihydroorotate dehydrogenase (DHODH) inhibitor DSM265 [60], and MMV 390048, a highly active anti-plasmodial compound which is a putative kinase inhibitor. Due to their high selectivity for parasite cells, these compounds were expected to be largely inactive. The cancer line panel comprised conventional commercially available cell lines as well as lines derived directly from patient-derived tumor xenografts (PDX). In order to gain insight in these molecules' mode of action we correlated their potency with the cancer lines' gene expression patterns. Finally we discuss our findings in the light of available pharmacokinetic data for these drug candidates and their clinical potential.

## Materials and Methods

### Tumor cell lines

The cell line panel comprised 68 cell lines derived from solid tumours and 24 cell lines derived from haematological malignancies [61]. Among the solid tumor cell lines, 30 were established at Oncotest from patient-derived tumor xenografts as described previously [61]–[63]. The other cell lines were either kindly provided by the NCI (Bethesda; MD) [64], or were purchased from ATCC (Rockville, MD), DSMZ (Braunschweig, Germany), ECACC (Salisbury, United Kingdom), KCLB (Seoul, Korea) or JCRB (Osaka, Japan). Authenticity of cell lines was verified at the DSMZ by STR (short tandem repeat) analysis, a PCR based DNA-fingerprinting methodology [65], [66]. Cell lines were routinely passaged once or twice weekly and maintained in culture for up to 20 passages. All cells were grown at 37°C in a humidified atmosphere with 5% CO_2_ in RPMI 1640 medium supplemented with 10% (v/v) fetal calf serum and 0.1 mg/mL gentamicin (medium and all components from PAA, Cölbe, Germany).

### Cell proliferation Assays

Cells were harvested from exponential phase cultures, counted and plated in 96 well flat-bottom microtiter plates at a cell density of 8,000–60,000 cells/well. After a 24 h recovery period to allow the cells to resume exponential growth, 10 µl of culture medium (six control wells/plate) or of culture medium with test compound(s) were added. The compounds were applied in duplicates at ten concentrations and treatment continued for four days. Determination of a potential synergism or antagonism by the median-effect principle of Chou-Talalay requires application of the combined substances at a constant, equipotent ratio, mostly at their IC_50_ values. Mixtures at this constant ratio were tested at ten different dilutions in duplicate. Additionally, each compound was also tested in monotherapy on the same plate. Both compounds were added simultaneously to the cells. Of each test combination, two to three independent experiments were performed.

For solid tumor derived cell lines, a modified propidium iodide (PI) assay [67] was used to assess the anti-cancer activity of the compounds. After the 4 four days treatment, cells were next washed with 200 µl PBS to remove dead cells, then 200 µl of a solution containing 7 µg/ml propidium iodide (PI) and 0.1% (v/v) Triton X-100 was added. After an incubation period of 1–2 hours at room temperature, fluorescence (FU) was measured using the Cytofluor® 4000 microplate reader (excitation λ = 530 nm, emission λ = 620 nm) to quantify the amount of attached viable cells.

For hematological cancer cell lines growing in suspension, the CellTiter-Blue® assay (#G8081, Promega) was used according to manufacturer's instructions. After treatment of cells, 10 µl/well CellTiter-Blue® reagent was added. Following an incubation period of up to four hours, fluorescence (FU) was measured by using the EnVision Xcite multilabel reader (excitation λ = 531 nm, emission λ = 615 nm).

For calculations, the mean value of duplicate/quadruplicate (untreated control) data was used. Calculation of IC_50_ values was done by 4 parameter non-linear curve fit (Oncotest Warehouse Software). The compounds were tested in 2–4 independent experiments and IC_50_ values are shown as the mean of those experiments.

### Assessment of drug-drug interaction (determination of CI according to Chou-Talalay)

Various two-drug combinations were evaluated using the median-effect principle proposed by Chou and Talalay [68]. This mathematical model, previously established for enzyme-substrate interactions, has been extended to multiple drug combinations. The formula of the median-effect principle is **f_a_/f_u_ = [D/D_m_]^m^**, in which f_a_ is the fraction of the cells affected, f_u_ (1-f_a_) is the fraction unaffected by the treatment, D the drug concentration, D_m_ the concentration required for 50% cell growth inhibition and m the slope of the median-effect curve (log[f_a_/f_u_] = m log D − m log (D_m_). The x-intercept yields log (D_m_), and thus, the D_m_ value. The deviation of data from the fitted median-effect equation is represented by the linear correlation coefficient r of the median-effect plot. Usually the experimental data from cell culture experiments have r >0.90.

The combination index (CI) was calculated by the Chou-Talalay equation, which takes into account both potency (D_m_ or IC_50_) and the shape of the dose-effect curve. The general equation for the classic isobologram (CI = 1) is given by: CI = (D)_1_/(D_x_)_1_ + (D)_2_(D_x_)_2_ + (D)_1_(D)_2_/(D_x_)_1_/(D_x_)_2_, where (D_x_)_1_ and (D_x_)_2_ in the denominators are the drug concentrations for D_1_ (drug 1) and D_2_ (drug 2) alone that gives x% inhibition, whereas (D)_1_ and (D)_2_ in the numerators are the concentrations of drug 1 and drug 2 in combination that also inhibited x% (i.e. isoeffective). CI<1, C = 1, and C>1 indicate synergism, additive effect, and antagonism, respectively. Calculations were performed with the computer program CalcuSyn developed by Chou and Hayball [69].

### Compounds

DHA was obtained from Chongqing Holley Wuling Mountain Pharmaceutical Co. Ltd, and artesunate from Guilin Pharmaceutical Co. Ltd.; artemisone, DSM265 [60], OZ277 [70], MMV390048 [71], OZ439 [72], OZ381 [73], P218 [59] and carbaOZ277 [74] were synthesized as previously described. Erlotinib and imatinib were supplied by LC-Laboratories (Woburn, MA, USA), cisplatin and vincristine by Sigma-Aldrich (Deisenhofen, Germany) and dasatinib by SanxinChemPharma (Shijiazhuang, China). Test compounds (antimalarials) were diluted in DMSO to a stock concentration of 26.7 mg/ml. Stock solutions for erlotinib, imatinib, cisplatin and dasatinib were prepared in DMSO at a concentration of 53.3 mM and vincristine at 67 µM.

### Microarrays

RNA was extracted from cell lines using the mirVana® kit (Ambion) according to the manufacturer's instructions. The RNA concentration was adjusted to 250 ng/µl. Biotinylated cRNA was prepared according to the standard Affymetrix protocol from total RNA (Expression Analysis Technical Manual, 2001, Affymetrix).

All microarray gene expression profiles were obtained using the Affymetrix HG-U133 Plus 2.0 GeneChip arrays. Hybridization was carried out according to standard Affymetrix protocol for 3′ IVT design and the GeneChips were scanned using the Hewlett-Packard GeneArray Scanner G2500A. All chips were assessed for quality using the “R” statistical computing environment and associated modules from Bioconductor with Percent Present cut-off of 35%, resulting in only a single array being rejected.

The CEL files were processed using RMAExpress (v. 1.04, Written by Ben Bolstad). The complete dataset (for 91 lines) was submitted to the Gene Expression Omnibus (GEO, http://www.ncbi.nlm.nih.gov/geo/query/acc.cgi?acc=GSE51739) under accession number GSE51739. Following background adjust, quantile normalization and Median Polish summarization, expression values were exported in natural scale for further analysis. Affymetrix probeset annotations for the HG-U133_Plus_2 microarray were taken from build 33 (29 Oct 2012). Using the Excel® CORREL (Pearson's) function, IC_50_s for each cell line were correlated with expression values, for a given compound and gene. In addition, probabilities for significance of these correlations were calculated using the Excel® formula  = TDIST(ABS([Correlation]*SQRT([nr of observations-2])/SQRT(1-[Correlation]∧2)), ([nr of observations-2],2). In this function, “Correlation” refers to correlation efficient r as calculated earlier, while “nr of observations” refers to the number of cell lines tested.

In order to subdivide sets of genes associated with compound potency into clusters with similar function, Gene Ontology Biological Process assignments (http://www.geneontology.org/) were taken from the Affymetrix annotation file. Frequency scoring and data processing were performed in Excel®. Cluster analysis was performed using the Excel add-in Multibase 2013 package (Numerical Dynamics, Japan), using the Ward method (Cluster membership is assessed by calculating the total sum of squared deviations from the mean of a cluster).

## Results

### Testing of antimalarials for activity against cancer cell lines

Antimalarials representing five distinct target classes were tested in this study: (*i*) the three artemisinins artesunate, dihydroartemisinin (Artenimol, DHA), and artemisone (BAY 44-9585), another semi-synthetic artemisinin derivative that presents an improved safety profile [57], [75]; (*ii*) synthetic ozonides OZ439 and 277, synthetic peroxides with enhanced *in vivo* stability [76]; (*iii*) pyrimethamine and P218, established and second-generation DHFR (dihydrofolate reductase) inhibitors, respectively [59]; (*iv*) DSM265, a potent and selective triazolopyrimidine-based inhibitor of the enzyme dihydroorotate dehydrogenase (DHODH) which kills Plasmodium *in vitro* and in an *in vivo* mouse model [60]; (*v*) MMV390048, a 3,5-diaryl-2-aminopyridine/2-trifluoromethylpyridine that is hypothesized to kill *Plasmodium* by inhibiting one or more of the parasite's kinases.

**Figure 1:**
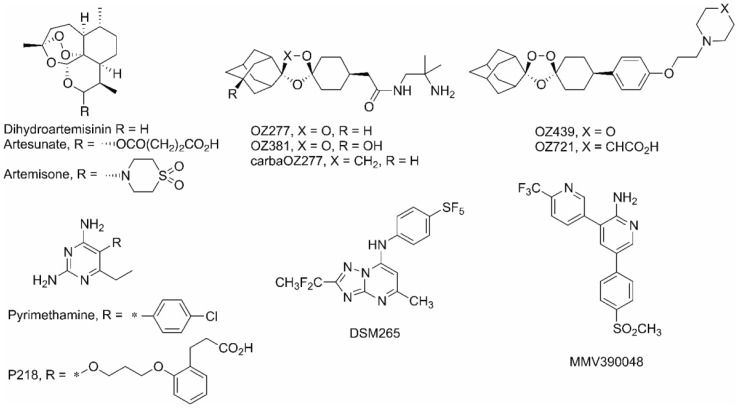
Molecular structures of compounds used in this study.

We included carbaOZ277, an inactive non-peroxide derivative of OZ 277 and OZ 381 and OZ 277, analogs of OZ277 and OZ439 (See Fig. 1 for their structures). As a positive control Paclitaxel, an established cancer drug, was included.

**Figure 2:**
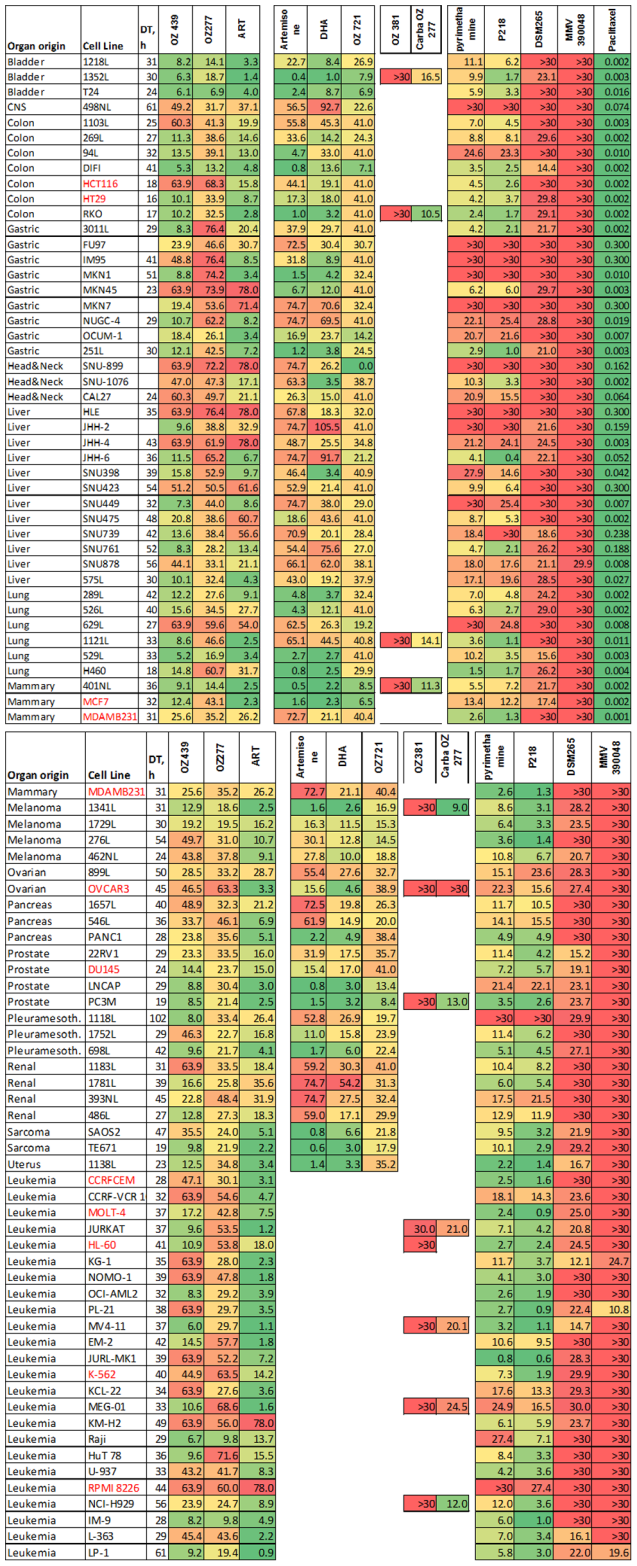
IC_50_s (in µM) of various antimalarials for a panel of human tumor cell lines. DT, h doubling time (hours).

Fig. 2 lists the potencies as IC_50_ (µM) for the set of solid tumors and leukemic cell lines tested, and the origin (organs) of the lines. There is considerable variation both between compounds and, for a given compound, between cell lines tested. In addition to paclitaxel, strong potencies were seen for the three artemisinins, the two synthetic peroxides and the two DHFR inhibitors. By contrast, the DHODH and assumed kinase inhibitors and negative control compounds (OZ381, OZ721, carbaOZ277) lacked antitumor activity in these assays. This is the first demonstration of an anticancer activity of synthetic peroxides. While a single study has demonstrated that pyrimethamine induces apoptosis in melanoma cells [58], we here extend these findings to a much larger panel of cancer types. In addition by using a second, different but related DHFR inhibitor, the fingerprint of specific inhibition can be defined. In some cases, lines that were relatively insensitive to paclitaxel also showed resistance to the antimalarials, especially the hepatoma lines (e.g. HLE, SNU423, SNU739). By contrast, other lines that responded poorly to paclitaxel showed excellent sensitivity towards the antimalarials (e.g. gastric cancer IM9).

### Effects of antimalarials on cancer cell expression profiles by microarray analysis

**Figure 3:**
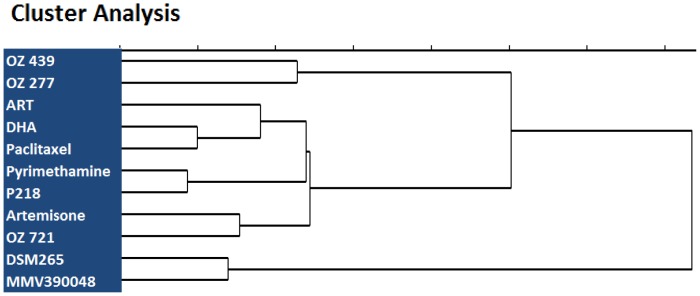
Cluster analysis for IC_50_s of various antimalarials. The IC_50_ stands for the compound concentration where half-maximal inhibition is observed.

In order to group the compounds tested on the basis of their relative potencies in this cell line panel we performed a cluster analysis, with the compound's IC_50_s as input for the MultiBase analytical package from Numerical Dynamics. As shown in Fig. 3, this analysis most tightly clustered the two synthetic peroxides and the two DHFR inhibitors. Surprisingly, paclitaxel clustered with ART, DHA and Artemisone. While artemisinins and paclitaxel each kill cancer cells by inducing apoptosis, the former (artemisinins) are believed to do so through the intrinsic apoptotic pathway involving caspase 3 and −9, whereas paclitaxel exploits the caspase 8 pathway [77]. The similarity we observe here could reflect that some of the cancer lines we have used are susceptible to apoptosis induced by either pathway. The fact that the synthetic peroxides cluster separately as a group, and are clearly separated from the artemisinins is intriguing. It suggests that the synthetic peroxides kill cancer cells by more than one mechanism in spite of the fact that they share a characteristic peroxide pharmacophore with the artemisinins. This also suggests that the mechanisms whereby these human cell lines develop resistance to artemisinins and to the synthetic peroxides could be completely different.

**Figure 4:**
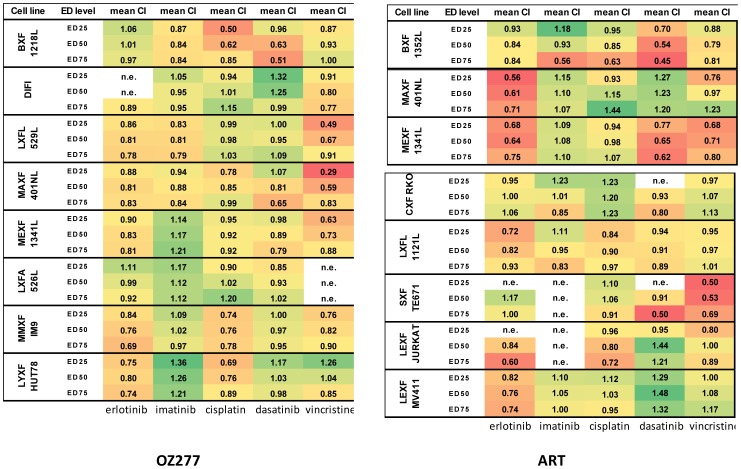
Assessment of drug-drug interaction (determination according to Chou-Talalay); A purely additive effect results in C = 1; lower Cl values reflect synergy (see Methods for details).

Since we found that the different antimalarials tested displayed distinct characteristics potentially reflecting diverse mode of actions we decided to further explore these compounds for potential synergies with the established anticancer drugs erlotinib, imatinib, cisplatin, dasatinib and vincristine. Using a subset of cell lines, the antimalarials were tested at three different concentrations together with a gradient of these anticancer drugs. The assessment of drug-drug interaction was determined according to Chou-Talalay (see Methods) and the results are displayed in Fig. 4. Both OZ 277 and ART exercised significant synergy with vincristine (as reflected by a low Cl value in the red-colored cells). Vincristine is known to polymerize microtubules, resulting in mitotic arrest in the metaphase, while earlier mitotic phases are unaffected [78]. By contrast, artemisinins are known to arrest cell division at a much earlier stage, namely the G1/G2 phase (eg. [56], [79]). Thus, while both type of compounds eventually induce apoptosis, it is possible that cells that somehow escape from the G1/2 phase block are subsequently trapped in the metaphase due to the action of vincristine, resulting in a synergetic mode of action.

Erlotinib is an EGFR (Epidermal growth factor receptor) inhibitor that we found synergizes with ART in five out of eight lines tested, while dasatinib, a c-abl inhibitor, stimulated activity in three lines. Since kinase inhibitors typically target a set of related kinases it may be difficult to predict which lines may present the best synergistic response.

**Figure 5:**
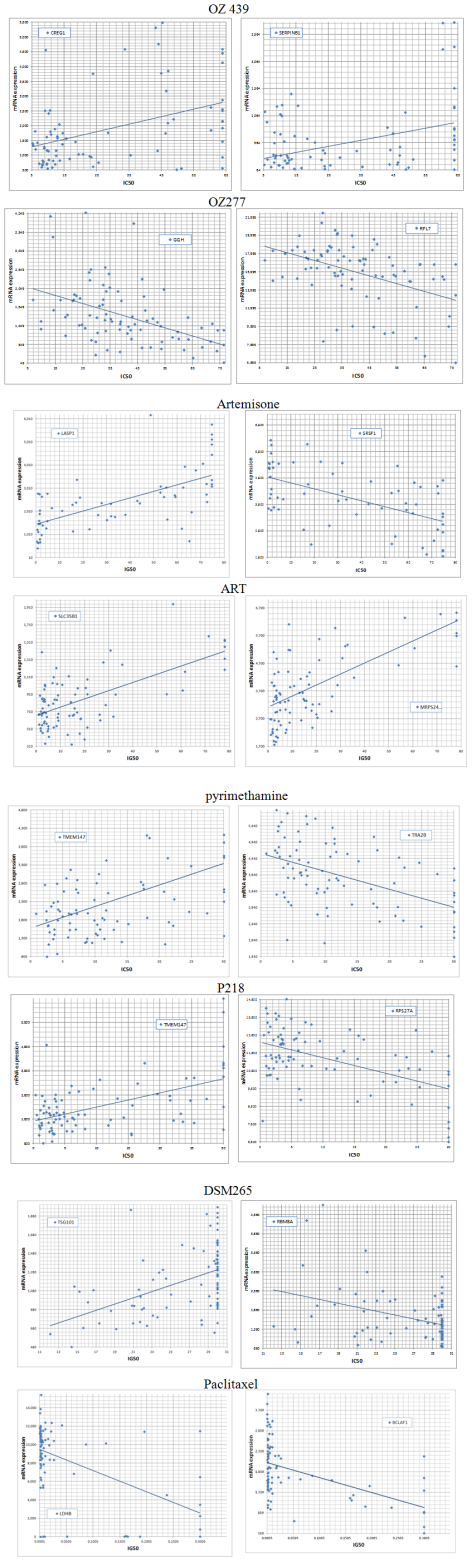
Relationship between gene expression across the cancer cell lines and IC50, for the indicated antimalarial and gene (HUGO code). CREG1: cellular repressor of E1A-stimulated genes 1, SERPINB1: serpin peptidase inhibitor, clade B (ovalbumin), member 1, GGH: γ-glutamyl, RPL7: ribosomal protein L7, LASP1: LIM and SH3 protein 1, SRSF1: serine/arginine-rich splicing factor 1, SLC35B1: solute carrier family 35, member B1, MRPS24: mitochondrial ribosomal protein, TMEM147: transmembrane protein 147, TRA2B: transformer 2 beta homolog (*Drosophila*), RPS27A: ribosomal protein S27a, TSG101: tumor susceptibility gene 101, RBM8A: RNA binding motif protein 8A, LDHB: lactate dehydrogenase B, BCLAF1: BCL2-associated transcription factor 1.

**Figure 6:**
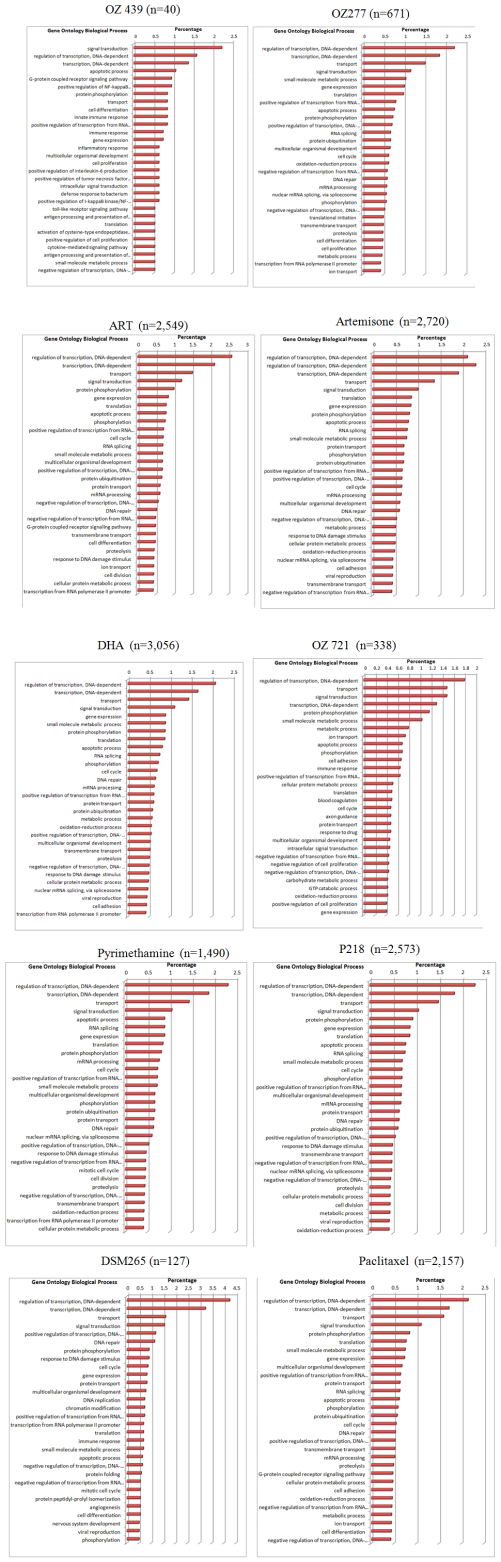
Predicted biological function (top 30) of genes whose expression is associated with IC_50_ (p<0.0005) for the various compounds. The histograms list biological processes that determine whether a drug will inhibit growth.

The checkered appearance of the Table in Fig. 2 reflects the modern view that cancers should be characterized by the molecular mechanism(s) that allows them to escape from proliferative controls, as opposed to a characterization by their organ of origin. In order to gain further insight in the molecular mechanisms that are associated with the compound's potencies we performed a gene expression microarray analysis of nearly every cell line used in this panel. For each compound tested, we subsequently correlated its inhibitory pattern throughout the cell line panel with gene expression variation, calculating correlation coefficients and their associated probabilities for each gene in the array (see Methods). We thus obtained, for each compound, lists of genes whose expression pattern correlated (positively or negatively) with compound potencies. Examples of such correlations are shown in Fig. 5. While expression of tollip (two probe sets) in most cell lines was somewhat below our 500 cutoff, potency of DHA is negatively correlated with expression of this gene (see Supplemental S1), a kinase substrate implicated in breast cancer [80]. A similar example is DUSP8, a gene whose promoter methylation predicts clinical outcome of ovarian cancer [81] and MAPKAPK2, which is known to regulate invasion of bladder cancer [82]. In order to obtain an aggregate, functional view of these associated genes we listed, for each compound, all genes whose expression significantly (cutoff p<0.0005) correlated with compound potency (positive and negative correlations were grouped). The corrected (Bonferroni) threshold for the 16,722 genes that we considered (those with expression signal >500) would be at p<3.10^−6^; however, this type of correction has been criticized as conservative (eg [83]) and might in our case skew the subsequent aggregate analysis (see below), which is less sensitive to the false discovery rate. The lists of genes associated with each compound are provided as a Supplement (S1). Next, the functional annotations (from the Gene Ontology Biological Process assignments, eg “DNA repair”) for the genes in each list were catalogued, and their frequencies (top 30) were plotted in histograms (Fig. 6), along with the number of genes for each list; similar results were obtained using the GENECODIS package ([84], data not shown). As expected, regulatory genes involved in signal transduction, transcription and apoptosis were prominent but, given that different gene sets associated with the compounds, remarkably similar; presumably, different genes associated with the various compounds were binned in the same category, resulting in the apparent convergence when results are displayed in this “high-level” format. Previously, a similar study to ours was performed with artesunate, but using a different cell line panel and microarray platform [85]. Interestingly, the top-ranked “resistance” gene in that study, SLC30A1, was also assigned by us as such (Data S1), and we found that expression of this gene was only significantly associated with ART (p = 0.0026), not the other compounds, emphasizing the unique character of each compound that we tested. SLC30A1 is a zinc efflux transporter; a *Plasmodium* orthologue exists with Swissprot accession Q8IBU1. One possibility that explains the confirmed link with ART resistance is that overexpression of zinc transporters protects from apoptosis [86]. The gene is also important in erythrocytes [87] and may affect the uptake of Fe^2+^ [88], which was shown to be a critical mediator of ART toxicity [89]–[92]. Another published study that evaluated ART and gene expression used the NCI60 cell line panel (from the National Institute for Cancer [64]). This study [90] identified the transferrin receptor (TRFC) as associated with ART resistance; in our study we also find this gene to be significantly associated with ART, artemisone and DHA (p = 0.01, 0.001 and 0.01, respectively) also as a “sensitivity gene” (i.e., higher expression results in greater compound sensitivity). From that study, we also confirmed the association of ART inhibition with ABC Transporter ABCB7 (p = 0.01).

Finally, a pathway analysis was performed to graphically display relationships between sets of genes in terms of the molecular partners they are known to interact with; using this type of analysis, separate sets of genes may reveal that they interact with a common “target” set of genes. However, an analysis using the VisANT package revealed no obvious relationships ([93], [94]; data not shown). One of the difficulties with this and similar packages (such as Ingenuity Pathway Analysis) is that “interactions” are derived from disparate observations: protein-protein interactions and enzyme-substrate relationship from different cell types and in various contexts. These relations are as yet incomplete and biased towards intensely studied and abundant (protein-protein interactions) partners.

## Discussion

We have evaluated five distinct classes of antimalarials for their potential use in cancer. Three of these, the artemisinins, the synthetic peroxides and DHFR (dihydrofolate reductase) inhibitors potently inhibited the growth of several human cancer lines. This high success rate may appear surprising, however, parasites and cancer cells share basic characteristics related to the metabolic requirements associated with their high proliferation rate. The parasiticidal and anticancer mechanisms of artemisinins have been extensively studied and are now well understood to be linked to an environment rich in free or heme-bound intracellular Fe^2+^ and the induction of apoptosis [13]. Nevertheless there are still uncertainties over the relative importance of the direct generation of reactive oxygen species and more indirect consequences of artemisinins binding to mitochondrial heme and the induction of the intrinsic apoptotic pathway. Our surprising finding that the three artemisinins can functionally be clearly distinguished from the synthetic peroxides, in spite of the fact that they all share a common peroxide pharmacophore, strongly suggests that these molecules have multiple modes of action, not all of which are distributed equally among members of the two families. DHFR is required for the mitochondrial thymidylate biosynthesis pathway, a critical process in rapidly reproducing cells. While the antiproliferative properties of these antifolates had mostly been evaluated in rapidly proliferating T lymphocytes, their potential use in cancer was only recently identified [58]. The present study confirms and extends these findings: we include a novel parasite DHFR inhibitor P218, that displays similar anticancer properties as pyrimethamine, and the similarity of the changes in the gene expression profiles of cell lines inhibited by the two compounds suggests a similarity in the mode of action. One of the reasons for developing P218 is the existing resistance observed in field isolates of *Plasmodium* to pyrimethamine. The similarity in the profiles of cancer cell lines inhibited by the two compounds, suggests that P218 may also be considered in combination with pyrimethamine to prevent the emergence of DHFR resistant tumor cells. We found no significant activity for DHODH inhibitors. One explanation for this is the stringent species specificity of these drugs (which target the *Plasmodium* ortholog), since one DHODH inhibitor, brequinar, did show (modest) efficacy in cancer [95]. The overarching pragmatic rationale for evaluating the activity of these antimalarials against cancer tissue-derived cell lines is that these drugs have well-established, excellent safety profiles. Furthermore, as we have shown in this study, their unique modes of action present opportunities for combinatorial use with established cancer drugs. Artemisinins are known to stall mitosis at the G1/G2 phase, while pyrimethamine results in a block at the S-phase [58] and paclitaxel at the metaphase; possibly such considerations constitute a firmer basis for deciding on potential for synergy between these drugs than a focus on the upstream pathways that these cancer cells utilize to drive proliferation. Furthermore, at least for the time being our descriptive capabilities (genetics and transcriptomics) have greatly outpaced our integrative capacity, in other words, our ability to reduce these data into an understanding of a cancer cell's specificity to cause disease. Either way, discovery of optimal combinatorial strategies remains therefore a matter of trial and error. In other words, while our data indicate that these compounds behave strikingly different, this knowledge cannot yet be translated into reliable predictions of how they should be used.

In order to assess the potential of these antimalarial compounds for a use in cancer chemotherapy it is also important to compare our data with their exposure in human subjects. Table 1 summarizes key pharmacokinetic properties for the active compounds used in this study, including C_max_, AUC (Area Under the Curve) and plasma half-life. Unfortunately these values are typically presented as weight/ml; as a rule of thumb and for comparison with the data in Fig. 2, 1 µg/ml corresponds to 2–3 µM (for compounds with MW 500 and 333, resp.). As the Table shows, artemisinins generally have a short plasma half-life. They are considered prodrugs with rapid conversion into DHA, which has a somewhat longer half-life. Since we show that both ART and its derivative DHA have anticancer properties, we can predict that efficacy may be better than one would assume from looking at exposure of either substance alone. Among the different artemisinin derivatives, artesunate is best suited for iv administration, providing a means for achieving the highest exposure. It is therefore presently the treatment of choice for acute malaria [96], [97] with peak exposure of 14–17 µg/ml when dosed at 120 mg iv [98], which is well above the IC_50_ for many of the cell lines that we tested (Fig. 2), even though the potencies that we found in our anticancer assays are much weaker than what is found when testing against *Plasmodium* (eg in the nM range for artesunate [75]). The short half-life of some of the compounds that we tested may be a problem for a mode of action that would involve inhibition of angiogenesis [21], but this is probably not a problem for antiproliferative uses (as against *Plasmodium*); successful anticancer drugs such as cisplatin and taxol also have rather short half-lives (1.5–3.6 and 5.8 h respectively). For the synthetic peroxides, exposure of the advanced second generation drug candidate OZ 439 reaches 2.4 µg/ml, or 5.1 µM [99]. This concentration brings the compound very close to the IC_50_s that we found for a number of cancer lines (Fig. 2). So far, this compound has been conservatively dosed in volunteers, commensurate with a use in malaria patients; however, its excellent safety profile could allow for significantly higher exposure in cancer patients. Also, in contrast to artemisinins the compound has excellent half-life properties, thus presenting considerable potential for clinical use. Finally, pyrimethamine generates an exposure of 2,059 ng/ml (∼8 µM; [100]), well above the IC_50_ of the majority of cell lines that we tested (Fig. 2). While the exposure in humans for P218 is not yet known, our study suggests that this compound has the potential to address resistance against this category of drugs both in malaria and cancer.

**Table 1:** Human pharmacokinetics of antimalarials tested in this study.

Compound (mg)	Cmax	AUC	Source	Half-life
**OZ 439 (capsule)**				
**50**	17 ng/ml	102 ng.h/ml	[99]	
**100**	34 ng/ml	249 ng.h/ml	[99]	
**200**	102 ng/ml	890 ng.h/ml	[99]	
**400**	135 ng/ml	1,130 ng.h/ml	[99]	
**800**	315 ng/ml	3,010 ng.h/ml	[99]	27.9 h
**1,200**	701 ng/ml	6,530 ng.h/ml	[99]	31.6 h
**OZ 439 (dispersion)**				
**400**	566 ng/ml	5,430 ng.h/ml	[99]	t1/2 = 31.2 h
**800**	917 ng/ml	9,630 ng.h/ml	[99]	t1/2 = 25.2 h
**1,600**	1,340 ng/ml	17,500 ng.h/ml	[99]	t1/2 = 30.7 h
**800**	∼2,400 ng/ml		[99]	
**800–1,200**	∼1,600 ng/ml		[99]	
**OZ 277**				
**50**	8 ng/ml	40 ng.h/ml (0–8 h)	[101]	
**100**	19 ng/ml	105 ng.h/ml	[101]	
**200**	41 ng/ml	239 ng.h/ml	[101]	
**50**	14 ng/ml	79 ng.h/ml (0–8 h)	[101]	
**100**	25 ng/ml	152 ng.h/ml	[101]	
**200**	68 ng/ml	408 ng.h/ml	[101]	
**ART/DHA**				
**120 mg iv**	13,700–17,000 ng/ml	876–1,038 ng.h/ml	[98]	2–3 min
**120 mg iv; DHA readout**	1,500–2,760 ng/ml	1,845–3,298 ng.h/ml	[98]	0.49–0.87 h
**200 mg po**	67–119 ng/ml	67–256 ng.h/ml	[98]	
**200 mg po, DHA readout**	654 ng/ml	1,158–1,300 ng.h/ml	[98]	
**120 mg im**	884 ng/ml	999 ng.h/ml	[98]	41 min
**120 mg im, DHA readout**	1,166 ng/ml	2,474 ng.h/ml	[98]	64 min
**120 mg ir**	448 ng/ml	796 ng.h/ml	[98]	0.95 h
**120 mg ir, DHA readout**	219 ng/ml	965 ng.h/ml	[98]	1.2 h
**40 mg DHA**	39 ng/ml	98 ng.h/ml (infin.)		
**60 mg ART**	183 ng/ml	155 ng.h/ml (infin.)		
**Artemisone**				
**10 mg po**	40 ng/ml	30 ng.h/ml	[102]	Up to 33.5 h
**20 mg po**	57 ng/ml	66 ng.h/ml	[102]	
**30 mg po**	51 ng/ml	72 ng.h/ml	[102]	
**40 mg po**	83 ng/ml	118 ng.h/ml	[102]	
**80 mg po**	140 ng/ml	282 ng.h/ml	[102]	
**pyrimethamine**				
**75 mg po**		34,700–38,400 ng.h/ml	[103]	
**50 mg/d, 3 wk**	2,059 ng/ml	41,800 ng/ml *h (24 h)	[100]	191 h
**75 mg single dose**	∼800 ng/ml	106,065 ng *h/ml	[104]	
**25 mg single dose**	760 ng/ml	76,000 ng *h/ml	[105]	114 h
**P218**				
**Rat 30 mg/kg**	15.8 uM (∼6,257 ng/ml)		[59]	7.3 h

In summary, we have established that three mechanistically different antimalarials show potent *in vitro* activity for a variety of cancer cell lines at concentrations that are established or reachable in patients. We also demonstrate evidence for potential synergy with established cancer drugs.

Given the well-established safety profile of these drugs, further evaluation of their potential in a clinical setting is recommended.

## Supporting Information

Data S1 **Correlation coefficients between gene expression and compound potency (cutoff p = 0.0005).** The “Minimum p-value” refers to the lowest p-value for the set of compounds that a gene was examined for.
(XLSX)

## References

[1] Krishna S, Bustamante L, Haynes RK, Staines HM (2008) Artemisinins: their growing importance in medicine. Trends Pharmacol Sci 29: 520–527. 18752857 10.1016/j.tips.2008.07.004 PMC2758403

[2] Zhang CZ, Pan Y, Cao Y, Lai PB, Liu L, et al (2012) Histone deacetylase inhibitors facilitate dihydroartemisinin-induced apoptosis in liver cancer in vitro and in vivo. PLoS One 7: e39870. 22761917 10.1371/journal.pone.0039870 PMC3386188

[3] Tin AS, Sundar SN, Tran KQ, Park AH, Poindexter KM, et al (2012) Antiproliferative effects of artemisinin on human breast cancer cells requires the downregulated expression of the E2F1 transcription factor and loss of E2F1-target cell cycle genes. Anticancer Drugs 23: 370–379. 22185819 10.1097/CAD.0b013e32834f6ea8

[4] Wang Z, Yu Y, Ma J, Zhang H, Zhang H, et al (2012) LyP-1 modification to enhance delivery of artemisinin or fluorescent probe loaded polymeric micelles to highly metastatic tumor and its lymphatics. Mol Pharm 9: 2646–2657. 22853186 10.1021/mp3002107

[5] Zhang CZ, Zhang H, Yun J, Chen GG, Lai PB (2012) Dihydroartemisinin exhibits antitumor activity toward hepatocellular carcinoma in vitro and in vivo. Biochem Pharmacol 83: 1278–1289. 22342732 10.1016/j.bcp.2012.02.002

[6] Soomro S, Langenberg T, Mahringer A, Konkimalla VB, Horwedel C, et al (2011) Design of novel artemisinin-like derivatives with cytotoxic and anti-angiogenic properties. J Cell Mol Med 15: 1122–1135. 20629994 10.1111/j.1582-4934.2010.01120.x PMC3822625

[7] Gao N, Budhraja A, Cheng S, Liu EH, Huang C, et al (2011) Interruption of the MEK/ERK signaling cascade promotes dihydroartemisinin-induced apoptosis in vitro and in vivo. Apoptosis 16: 511–523. 21336837 10.1007/s10495-011-0580-6

[8] Noori S, Hassan ZM (2011) Dihydroartemisinin shift the immune response towards Th1, inhibit the tumor growth in vitro and in vivo. Cell Immunol 271: 67–72. 21820106 10.1016/j.cellimm.2011.06.008

[9] Singh NP, Lai HC, Park JS, Gerhardt TE, Kim BJ, et al (2011) Effects of artemisinin dimers on rat breast cancer cells in vitro and in vivo. Anticancer Res 31: 4111–4114. 22199268

[10] Weifeng T, Feng S, Xiangji L, Changqing S, Zhiquan Q, et al (2011) Artemisinin inhibits in vitro and in vivo invasion and metastasis of human hepatocellular carcinoma cells. Phytomedicine 18: 158–162. 20739158 10.1016/j.phymed.2010.07.003

[11] Zhao Y, Jiang W, Li B, Yao Q, Dong J, et al (2011) Artesunate enhances radiosensitivity of human non-small cell lung cancer A549 cells via increasing NO production to induce cell cycle arrest at G2/M phase. Int Immunopharmacol 11: 2039–2046. 21907831 10.1016/j.intimp.2011.08.017

[12] Xu Q, Li ZX, Peng HQ, Sun ZW, Cheng RL, et al (2011) Artesunate inhibits growth and induces apoptosis in human osteosarcoma HOS cell line in vitro and in vivo. J Zhejiang Univ Sci B 12: 247–255. 21462379 10.1631/jzus.B1000373 PMC3072588

[13] Crespo-Ortiz MP, Wei MQ (2012) Antitumor activity of artemisinin and its derivatives: from a well-known antimalarial agent to a potential anticancer drug. J Biomed Biotechnol 2012: 247597. 22174561 10.1155/2012/247597 PMC3228295

[14] Zhang ZY, Yu SQ, Miao LY, Huang XY, Zhang XP, et al (2008) [Artesunate combined with vinorelbine plus cisplatin in treatment of advanced non-small cell lung cancer: a randomized controlled trial]. Zhong Xi Yi Jie He Xue Bao 6: 134–138. 18241646 10.3736/jcim20080206

[15] Singh NP, Panwar VK (2006) Case report of a pituitary macroadenoma treated with artemether. Integr Cancer Ther 5: 391–394. 17101767 10.1177/1534735406295311

[16] Berger TG, Dieckmann D, Efferth T, Schultz ES, Funk JO, et al (2005) Artesunate in the treatment of metastatic uveal melanoma–first experiences. Oncol Rep 14: 1599–1603. 16273263

[17] Panossian LA, Garga NI, Pelletier D (2005) Toxic brainstem encephalopathy after artemisinin treatment for breast cancer. Ann Neurol 58: 812–813. 16240360 10.1002/ana.20620

[18] Singh NP, Verma KB (2002) Case report of a laryngeal squamous cell carcinoma treated with artesunate. Archive of Oncology 10: 279–280.

[19] Rowen RJ (2002) Chinese Herb Cures Cancer. Second Opinion XII.

[20] White CL (2002) Cancer Smart Bomb, Part I: An Idea from Ancient Chinese Medicine. Excerpted from New Horizons, Summer 2002 issue Brewer Science Library.

[21] Li Q, Weina P, Hickma M (2013) The Use of Artemisinin Compounds as Angiogenesis Inhibitors to Treat Cancer. In: Chai J, editor. Research Directions in Tumor Angiogenesis.

[22] Byakika-Kibwika P, Lamorde M, Mayito J, Nabukeera L, Mayanja-Kizza H, et al (2012) Pharmacokinetics and pharmacodynamics of intravenous artesunate during severe malaria treatment in Ugandan adults. Malar J 11: 132. 22540954 10.1186/1475-2875-11-132 PMC3489518

[23] Newton PN, Barnes KI, Smith PJ, Evans AC, Chierakul W, et al (2006) The pharmacokinetics of intravenous artesunate in adults with severe falciparum malaria. Eur J Clin Pharmacol 62: 1003–1009. 17089111 10.1007/s00228-006-0203-2

[24] Khanh NX, de Vries PJ, Ha LD, van Boxtel CJ, Koopmans R, et al (1999) Declining concentrations of dihydroartemisinin in plasma during 5-day oral treatment with artesunate for Falciparum malaria. Antimicrob Agents Chemother 43: 690–692. 10049291 10.1128/aac.43.3.690 PMC89184

[25] Ashton M, Hai TN, Sy ND, Huong DX, Van Huong N, et al (1998) Artemisinin pharmacokinetics is time-dependent during repeated oral administration in healthy male adults. Drug Metab Dispos 26: 25–27. 9443848

[26] Hien TT, White NJ (1993) Qinghaosu. Lancet 341: 603–608. 8094838 10.1016/0140-6736(93)90362-k

[27] Zhang S, Gerhard GS (2008) Heme activates artemisinin more efficiently than hemin, inorganic iron, or hemoglobin. Bioorg Med Chem 16: 7853–7861. 18676152 10.1016/j.bmc.2008.02.034

[28] Meshnick SR, Thomas A, Ranz A, Xu CM, Pan HZ (1991) Artemisinin (qinghaosu): the role of intracellular hemin in its mechanism of antimalarial action. Mol Biochem Parasitol 49: 181–189. 1775162 10.1016/0166-6851(91)90062-b

[29] O'Neill PM, Barton VE, Ward SA (2010) The molecular mechanism of action of artemisinin–the debate continues. Molecules 15: 1705–1721. 20336009 10.3390/molecules15031705 PMC6257357

[30] Noori S, Hassan ZM, Taghikhani M, Rezaei B, Habibi Z (2010) Dihydroartemisinin can inhibit calmodulin, calmodulin-dependent phosphodiesterase activity and stimulate cellular immune responses. Int Immunopharmacol 10: 213–217. 19900584 10.1016/j.intimp.2009.11.002

[31] Chen X, Lin H, Wen J, Rong Q, Xu W, et al (2012) Dihydroartemisinin suppresses cell proliferation, invasion, and angiogenesis in human glioma U87 cells. African Journal of Pharmacy and Pharmacology 6: 2433–2440.

[32] Eichhorn T, Schloissnig S, Hahn B, Wendler A, Mertens R, et al (2012) Bioinformatic and experimental fishing for artemisinin-interacting proteins from human nasopharyngeal cancer cells. Mol Biosyst 8: 1311–1318. 22311186 10.1039/c2mb05437j

[33] Chen H, Shi L, Yang X, Li S, Guo X, et al (2010) Artesunate inhibiting angiogenesis induced by human myeloma RPMI8226 cells. Int J Hematol 92: 587–597. 20945119 10.1007/s12185-010-0697-3

[34] Zhou HJ, Wang WQ, Wu GD, Lee J, Li A (2007) Artesunate inhibits angiogenesis and downregulates vascular endothelial growth factor expression in chronic myeloid leukemia K562 cells. Vascul Pharmacol 47: 131–138. 17581794 10.1016/j.vph.2007.05.002

[35] Wang J, Guo Y, Zhang BC, Chen ZT, Gao JF (2007) Induction of apoptosis and inhibition of cell migration and tube-like formation by dihydroartemisinin in murine lymphatic endothelial cells. Pharmacology 80: 207–218. 17622766 10.1159/000104418

[36] Chen HH, Zhou HJ, Wu GD, Lou XE (2004) Inhibitory effects of artesunate on angiogenesis and on expressions of vascular endothelial growth factor and VEGF receptor KDR/flk-1. Pharmacology 71: 1–9. 15051917 10.1159/000076256

[37] Huan-huan C, Li-Li Y, Shang-Bin L (2004) Artesunate reduces chicken chorioallantoic membrane neovascularisation and exhibits antiangiogenic and apoptotic activity on human microvascular dermal endothelial cell. Cancer Lett 211: 163–173. 15219940 10.1016/j.canlet.2004.03.014

[38] Zhou C, Pan W, Wang XP, Chen TS (2012) Artesunate induces apoptosis via a Bak-mediated caspase-independent intrinsic pathway in human lung adenocarcinoma cells. J Cell Physiol 227: 3778–3786. 22378505 10.1002/jcp.24086

[39] Handrick R, Ontikatze T, Bauer KD, Freier F, Rubel A, et al (2010) Dihydroartemisinin induces apoptosis by a Bak-dependent intrinsic pathway. Mol Cancer Ther 9: 2497–2510. 20663933 10.1158/1535-7163.MCT-10-0051

[40] Kim SJ, Kim MS, Lee JW, Lee CH, Yoo H, et al (2006) Dihydroartemisinin enhances radiosensitivity of human glioma cells in vitro. J Cancer Res Clin Oncol 132: 129–135. 16273420 10.1007/s00432-005-0052-x PMC12161046

[41] Singh NP, Lai HC (2004) Artemisinin induces apoptosis in human cancer cells. Anticancer Res 24: 2277–2280. 15330172

[42] Efferth T, Benakis A, Romero MR, Tomicic M, Rauh R, et al (2004) Enhancement of cytotoxicity of artemisinins toward cancer cells by ferrous iron. Free Radic Biol Med 37: 998–1009. 15336316 10.1016/j.freeradbiomed.2004.06.023

[43] Sadava D, Phillips T, Lin C, Kane SE (2002) Transferrin overcomes drug resistance to artemisinin in human small-cell lung carcinoma cells. Cancer Lett 179: 151–156. 11888669 10.1016/s0304-3835(02)00005-8

[44] Lai H, Singh NP (1995) Selective cancer cell cytotoxicity from exposure to dihydroartemisinin and holotransferrin. Cancer Lett 91: 41–46. 7750093 10.1016/0304-3835(94)03716-v

[45] Daniels TR, Delgado T, Helguera G, Penichet ML (2006) The transferrin receptor part II: targeted delivery of therapeutic agents into cancer cells. Clin Immunol 121: 159–176. 16920030 10.1016/j.clim.2006.06.006

[46] Daniels TR, Delgado T, Rodriguez JA, Helguera G, Penichet ML (2006) The transferrin receptor part I: Biology and targeting with cytotoxic antibodies for the treatment of cancer. Clin Immunol 121: 144–158. 16904380 10.1016/j.clim.2006.06.010

[47] Zeng QP, Zhang PZ (2011) Artesunate mitigates proliferation of tumor cells by alkylating heme-harboring nitric oxide synthase. Nitric Oxide 24: 110–112. 21168521 10.1016/j.niox.2010.12.005

[48] Zhang S, Gerhard GS (2009) Heme mediates cytotoxicity from artemisinin and serves as a general anti-proliferation target. PLoS One 4: e7472. 19862332 10.1371/journal.pone.0007472 PMC2764339

[49] Li W, Mo W, Shen D, Sun L, Wang J, et al (2005) Yeast model uncovers dual roles of mitochondria in action of artemisinin. PLoS Genet 1: e36. 16170412 10.1371/journal.pgen.0010036 PMC1201371

[50] Huang XJ, Li CT, Zhang WP, Lu YB, Fang SH, et al (2008) Dihydroartemisinin potentiates the cytotoxic effect of temozolomide in rat C6 glioma cells. Pharmacology 82: 1–9. 18408414 10.1159/000125673

[51] Efferth T, Giaisi M, Merling A, Krammer PH, Li-Weber M (2007) Artesunate induces ROS-mediated apoptosis in doxorubicin-resistant T leukemia cells. PLoS One 2: e693. 17668070 10.1371/journal.pone.0000693 PMC1933253

[52] Kong R, Jia G, Cheng ZX, Wang YW, Mu M, et al (2012) Dihydroartemisinin enhances Apo2L/TRAIL-mediated apoptosis in pancreatic cancer cells via ROS-mediated up-regulation of death receptor 5. PLoS One 7: e37222. 22666346 10.1371/journal.pone.0037222 PMC3364248

[53] Lu JJ, Chen SM, Ding J, Meng LH (2012) Characterization of dihydroartemisinin-resistant colon carcinoma HCT116/R cell line. Mol Cell Biochem 360: 329–337. 21959972 10.1007/s11010-011-1072-2

[54] Efferth T, Herrmann F, Tahrani A, Wink M (2011) Cytotoxic activity of secondary metabolites derived from Artemisia annua L. towards cancer cells in comparison to its designated active constituent artemisinin. Phytomedicine 18: 959–969. 21831619 10.1016/j.phymed.2011.06.008

[55] Anfosso L, Efferth T, Albini A, Pfeffer U (2006) Microarray expression profiles of angiogenesis-related genes predict tumor cell response to artemisinins. Pharmacogenomics J 6: 269–278. 16432535 10.1038/sj.tpj.6500371

[56] Efferth T, Dunstan H, Sauerbrey A, Miyachi H, Chitambar CR (2001) The anti-malarial artesunate is also active against cancer. Int J Oncol 18: 767–773. 11251172 10.3892/ijo.18.4.767

[57] Haynes RK, Fugmann B, Stetter J, Rieckmann K, Heilmann HD, et al (2006) Artemisone–a highly active antimalarial drug of the artemisinin class. Angew Chem Int Ed Engl 45: 2082–2088. 16444785 10.1002/anie.200503071

[58] Giammarioli AM, Maselli A, Casagrande A, Gambardella L, Gallina A, et al (2008) Pyrimethamine induces apoptosis of melanoma cells via a caspase and cathepsin double-edged mechanism. Cancer Res 68: 5291–5300. 18593930 10.1158/0008-5472.CAN-08-0222

[59] Yuthavong Y, Tarnchompoo B, Vilaivan T, Chitnumsub P, Kamchonwongpaisan S, et al (2012) Malarial dihydrofolate reductase as a paradigm for drug development against a resistance-compromised target. Proc Natl Acad Sci U S A 109: 16823–16828. 23035243 10.1073/pnas.1204556109 PMC3479511

[60] Coteron JM, Marco M, Esquivias J, Deng X, White KL, et al (2011) Structure-guided lead optimization of triazolopyrimidine-ring substituents identifies potent Plasmodium falciparum dihydroorotate dehydrogenase inhibitors with clinical candidate potential. J Med Chem 54: 5540–5561. 21696174 10.1021/jm200592f PMC3156099

[61] Roth T, Burger AM, Dengler W, Willmann H, Fiebig HH (1999) Human tumor cell lines demonstrating the characteristics of patient tumors as useful models for anticancer drug screening. In: Fiebig HH, Burger AM, editors. Relevance of Tumor Models for Anticancer Drug Development, Contrib Oncol pp. 145–156.

[62] Fiebig HH, Winterhalter B, Berger DP, Lohr GW (1989) Combined in vitro/in vivo test procedure with human tumor xenografts for anticancer drug development. Strahlenther Onkol 165: 522–524. 2749489

[63] Fiebig HH, Dengler WA, Roth T (1999) Human tumor xenografts: Predictivity, characterization, and discovery of new anticancer agents. In: Fiebig HH, Burger AM, editors. Relevance of Tumor Models for Anticancer Drug Development Contrib Oncol. pp. 29–50.

[64] Shoemaker RH (2006) The NCI60 human tumour cell line anticancer drug screen. Nat Rev Cancer 6: 813–823. 16990858 10.1038/nrc1951

[65] Masters JR, Thomson JA, Daly-Burns B, Reid YA, Dirks WG, et al (2001) Short tandem repeat profiling provides an international reference standard for human cell lines. Proc Natl Acad Sci U S A 98: 8012–8017. 11416159 10.1073/pnas.121616198 PMC35459

[66] Dirks WG, Faehnrich S, Estella IA, Drexler HG (2005) Short tandem repeat DNA typing provides an international reference standard for authentication of human cell lines. ALTEX 22: 103–109. 15953965

[67] Dengler WA, Schulte J, Berger DP, Mertelsmann R, Fiebig HH (1995) Development of a propidium iodide fluorescence assay for proliferation and cytotoxicity assays. Anticancer Drugs 6: 522–532. 7579556 10.1097/00001813-199508000-00005

[68] Chou TC (1991) The median-effect principle and the combination index for quantitation of synergism and antagonism. In: Chou TC, Rideout DC, editors. Synergism and antagonism in chemotherapy. San Diego: Academic Press. pp. 61–102.

[69] Chou TC, Hayball MP (1996) CalcuSyn for Windows. Software for Dose Effect Analysis. Cambridge: Biosoft Copyright 1996–2005 (CalcuSyn 2).

[70] Vennerstrom JL, Arbe-Barnes S, Brun R, Charman SA, Chiu FC, et al (2004) Identification of an antimalarial synthetic trioxolane drug development candidate. Nature 430: 900–904. 15318224 10.1038/nature02779

[71] Younis Y, Douelle F, Feng TS, Gonzalez Cabrera D, Le Manach C, et al (2012) 3,5-Diaryl-2-aminopyridines as a novel class of orally active antimalarials demonstrating single dose cure in mice and clinical candidate potential. J Med Chem 55: 3479–3487. 22390538 10.1021/jm3001373

[72] Charman SA, Arbe-Barnes S, Bathurst IC, Brun R, Campbell M, et al (2011) Synthetic ozonide drug candidate OZ439 offers new hope for a single-dose cure of uncomplicated malaria. Proc Natl Acad Sci U S A 108: 4400–4405. 21300861 10.1073/pnas.1015762108 PMC3060245

[73] Zhou L, Alker A, Ruf A, Wang X, Chiu FC, et al (2008) Characterization of the two major CYP450 metabolites of ozonide (1,2,4-trioxolane) OZ277. Bioorg Med Chem Lett 18: 1555–1558. 18262417 10.1016/j.bmcl.2008.01.087

[74] Kaiser M, Wittlin S, Nehrbass-Stuedli A, Dong Y, Wang X, et al (2007) Peroxide bond-dependent antiplasmodial specificity of artemisinin and OZ277 (RBx11160). Antimicrob Agents Chemother 51: 2991–2993. 17562801 10.1128/AAC.00225-07 PMC1932508

[75] Ramharter M, Burkhardt D, Nemeth J, Adegnika AA, Kremsner PG (2006) In vitro activity of artemisone compared with artesunate against Plasmodium falciparum. Am J Trop Med Hyg 75: 637–639. 17038685

[76] Wang X, Dong Y, Wittlin S, Charman SA, Chiu FC, et al (2013) Comparative Antimalarial Activities and ADME Profiles of Ozonides (1,2,4-trioxolanes) OZ277, OZ439, and Their 1,2-Dioxolane, 1,2,4-Trioxane, and 1,2,4,5-Tetraoxane Isosteres. J Med Chem 10.1021/jm400004u 23489135

[77] Mielgo A, Torres VA, Clair K, Barbero S, Stupack DG (2009) Paclitaxel promotes a caspase 8-mediated apoptosis through death effector domain association with microtubules. Oncogene 28: 3551–3562. 19668227 10.1038/onc.2009.210 PMC2851247

[78] Wagner HP, Swidzinska PM, Hirt A (1977) Variable duration of vincristine-induced metaphase block in leukemic and nornal bone marrow cells of children. Med Pediatr Oncol 3: 75–83. 265000 10.1002/mpo.2950030111

[79] Chen H, Sun B, Wang S, Pan S, Gao Y, et al (2010) Growth inhibitory effects of dihydroartemisinin on pancreatic cancer cells: involvement of cell cycle arrest and inactivation of nuclear factor-kappaB. J Cancer Res Clin Oncol 136: 897–903. 19941148 10.1007/s00432-009-0731-0 PMC11828290

[80] Chen Y, Choong LY, Lin Q, Philp R, Wong CH, et al (2007) Differential expression of novel tyrosine kinase substrates during breast cancer development. Mol Cell Proteomics 6: 2072–2087. 17855441 10.1074/mcp.M700395-MCP200

[81] Lim S, Green JA, Wong H, VanderBurg ME, Crook T (2007) DUSP7 and DUSP8 promoter hypermethylations: Predictors of clinical outcomes in advanced epithelial ovarian carcinoma. Journal of Clinical Oncology 25: 5501.

[82] Kumar B, Koul S, Petersen J, Khandrika L, Hwa JS, et al (2010) p38 mitogen-activated protein kinase-driven MAPKAPK2 regulates invasion of bladder cancer by modulation of MMP-2 and MMP-9 activity. Cancer Res 70: 832–841. 20068172 10.1158/0008-5472.CAN-09-2918

[83] Perneger TV (1998) What's wrong with Bonferroni adjustments. BMJ 316: 1236–1238. 9553006 10.1136/bmj.316.7139.1236 PMC1112991

[84] Carmona-Saez P, Chagoyen M, Tirado F, Carazo JM, Pascual-Montano A (2007) GENECODIS: a web-based tool for finding significant concurrent annotations in gene lists. Genome Biol 8: R3. 17204154 10.1186/gb-2007-8-1-r3 PMC1839127

[85] Sertel S, Eichhorn T, Simon CH, Plinkert PK, Johnson SW, et al (2010) Pharmacogenomic identification of c-Myc/Max-regulated genes associated with cytotoxicity of artesunate towards human colon, ovarian and lung cancer cell lines. Molecules 15: 2886–2910. 20428086 10.3390/molecules15042886 PMC6257326

[86] Zhang X, Liang D, Guo B, Deng W, Chi ZH, et al (2013) Zinc transporter 5 and zinc transporter 7 induced by high glucose protects peritoneal mesothelial cells from undergoing apoptosis. Cell Signal 25: 999–1010. 23275032 10.1016/j.cellsig.2012.12.013

[87] Ryu MS, Guthrie GJ, Maki AB, Aydemir TB, Cousins RJ (2012) Proteomic analysis shows the upregulation of erythrocyte dematin in zinc-restricted human subjects. Am J Clin Nutr 95: 1096–1102. 22456662 10.3945/ajcn.111.032862 PMC3325834

[88] Iyengar V, Pullakhandam R, Nair KM (2012) Coordinate expression and localization of iron and zinc transporters explain iron-zinc interactions during uptake in Caco-2 cells: implications for iron uptake at the enterocyte. J Nutr Biochem 23: 1146–1154. 22137264 10.1016/j.jnutbio.2011.06.008

[89] Lu JJ, Meng LH, Cai YJ, Chen Q, Tong LJ, et al (2008) Dihydroartemisinin induces apoptosis in HL-60 leukemia cells dependent of iron and p38 mitogen-activated protein kinase activation but independent of reactive oxygen species. Cancer Biol Ther 7: 1017–1023. 18414062 10.4161/cbt.7.7.6035

[90] Kelter G, Steinbach D, Konkimalla VB, Tahara T, Taketani S, et al (2007) Role of transferrin receptor and the ABC transporters ABCB6 and ABCB7 for resistance and differentiation of tumor cells towards artesunate. PLoS One 2: e798. 17726528 10.1371/journal.pone.0000798 PMC1949049

[91] Huang XJ, Ma ZQ, Zhang WP, Lu YB, Wei EQ (2007) Dihydroartemisinin exerts cytotoxic effects and inhibits hypoxia inducible factor-1alpha activation in C6 glioma cells. J Pharm Pharmacol 59: 849–856. 17637177 10.1211/jpp.59.6.0011

[92] Zhou HJ, Wang Z, Li A (2008) Dihydroartemisinin induces apoptosis in human leukemia cells HL60 via downregulation of transferrin receptor expression. Anticancer Drugs 19: 247–255. 18510170 10.1097/cad.0b013e3282f3f152

[93] Hu Z, Hung JH, Wang Y, Chang YC, Huang CL, et al (2009) VisANT 3.5: multi-scale network visualization, analysis and inference based on the gene ontology. Nucleic Acids Res 37: W115–121. 19465394 10.1093/nar/gkp406 PMC2703932

[94] Hu Z, Mellor J, Wu J, DeLisi C (2004) VisANT: an online visualization and analysis tool for biological interaction data. BMC Bioinformatics 5: 17. 15028117 10.1186/1471-2105-5-17 PMC368431

[95] Cody R, Stewart D, DeForni M, Moore M, Dallaire B, et al (1993) Multicenter phase II study of brequinar sodium in patients with advanced breast cancer. Am J Clin Oncol 16: 526–528. 8256771 10.1097/00000421-199312000-00014

[96] Dondorp A, Nosten F, Stepniewska K, Day N, White N, et al (2005) Artesunate versus quinine for treatment of severe falciparum malaria: a randomised trial. Lancet 366: 717–725. 16125588 10.1016/S0140-6736(05)67176-0

[97] Dondorp AM, Fanello CI, Hendriksen IC, Gomes E, Seni A, et al (2010) Artesunate versus quinine in the treatment of severe falciparum malaria in African children (AQUAMAT): an open-label, randomised trial. Lancet 376: 1647–1657. 21062666 10.1016/S0140-6736(10)61924-1 PMC3033534

[98] Morris CA, Duparc S, Borghini-Fuhrer I, Jung D, Shin CS, et al (2011) Review of the clinical pharmacokinetics of artesunate and its active metabolite dihydroartemisinin following intravenous, intramuscular, oral or rectal administration. Malar J 10: 263. 21914160 10.1186/1475-2875-10-263 PMC3180444

[99] Moehrle JJ, Duparc S, Siethoff C, van Giersbergen PL, Craft JC, et al (2012) First-in-man safety and pharmacokinetics of synthetic ozonide OZ439 demonstrates an improved exposure profile relative to other peroxide antimalarials. Br J Clin Pharmacol 10.1111/j.1365-2125.2012.04368.x PMC3558805 22759078

[100] Jacobson JM, Davidian M, Rainey PM, Hafner R, Raasch RH, et al (1996) Pyrimethamine pharmacokinetics in human immunodeficiency virus-positive patients seropositive for Toxoplasma gondii. Antimicrob Agents Chemother 40: 1360–1365. 8726001 10.1128/aac.40.6.1360 PMC163331

[101] Valecha N, Looareesuwan S, Martensson A, Abdulla SM, Krudsood S, et al (2010) Arterolane, a new synthetic trioxolane for treatment of uncomplicated *Plasmodium falciparum* malaria: a phase II, multicenter, randomized, dose-finding clinical trial. Clin Infect Dis 51: 684–691. 20687837 10.1086/655831

[102] Nagelschmitz J, Voith B, Wensing G, Roemer A, Fugmann B, et al (2008) First assessment in humans of the safety, tolerability, pharmacokinetics, and ex vivo pharmacodynamic antimalarial activity of the new artemisinin derivative artemisone. Antimicrob Agents Chemother 52: 3085–3091. 18559649 10.1128/AAC.01585-07 PMC2533467

[103] Green MD, van Eijk AM, van Ter Kuile FO, Ayisi JG, Parise ME, et al (2007) Pharmacokinetics of sulfadoxine-pyrimethamine in HIV-infected and uninfected pregnant women in Western Kenya. J Infect Dis 196: 1403–1408. 17922406 10.1086/522632

[104] Karunajeewa HA, Salman S, Mueller I, Baiwog F, Gomorrai S, et al (2009) Pharmacokinetic properties of sulfadoxine-pyrimethamine in pregnant women. Antimicrob Agents Chemother 53: 4368–4376. 19620325 10.1128/AAC.00335-09 PMC2764169

[105] Mansor SM, Navaratnam V, Mohamad M, Hussein S, Kumar A, et al (1989) Single dose kinetic study of the triple combination mefloquine/sulphadoxine/pyrimethamine (Fansimef) in healthy male volunteers. Br J Clin Pharmacol 27: 381–386. 2785812 10.1111/j.1365-2125.1989.tb05381.x PMC1379839

